# Silicone encapsulation of thin-film SiO_*x*_, SiO_*x*_N_*y*_ and SiC for modern electronic medical implants: a comparative long-term ageing study

**DOI:** 10.1088/1741-2552/abf0d6

**Published:** 2021-04-06

**Authors:** C Lamont, T Grego, K Nanbakhsh, A Shah Idil, V Giagka, A Vanhoestenberghe, S Cogan, N Donaldson

**Affiliations:** 1 Implanted Devices Group, Department of Medical Physics and Biomedical Engineering, University College London, Gower Street, London, United Kingdom; 2 Department of Microelectronics, Faculty of Electrical Engineering, Mathematics and Computer Science, Delft University of Technology, Delft, The Netherlands; 3 Institute of Orthopaedics and Musculoskeletal Science, RNOH Trust, University College London, Stanmore, United Kingdom; 4 Department of System Integration and Interconnection Technologies, Fraunhofer Institute for Reliability and Micro-integration IZM, Berlin, Germany; 5 Department of Bioengineering, University of Texas at Dallas, Richardson, TX, United States of America

**Keywords:** accelerated tests, electrochemical impedance spectroscopy, encapsulation, impedance spectroscopy, integrated circuit, life-testing, silicone

## Abstract

*Objective.* Ensuring the longevity of implantable devices is critical for their clinical usefulness. This is commonly achieved by hermetically sealing the sensitive electronics in a water impermeable housing, however, this method limits miniaturisation. Alternatively, silicone encapsulation has demonstrated long-term protection of implanted thick-film electronic devices. However, much of the current conformal packaging research is focused on more rigid coatings, such as parylene, liquid crystal polymers and novel inorganic layers. Here, we consider the potential of silicone to protect implants using thin-film technology with features 33 times smaller than thick-film counterparts. *Approach.* Aluminium interdigitated comb structures under plasma-enhanced chemical vapour deposited passivation (SiO_*x*_, SiO_*x*_N_*y*_, SiO_*x*_N_*y*_ + SiC) were encapsulated in medical grade silicones, with a total of six passivation/silicone combinations. Samples were aged in phosphate-buffered saline at 67 ^∘^C for up to 694 days under a continuous ±5 V biphasic waveform. Periodic electrochemical impedance spectroscopy measurements monitored for leakage currents and degradation of the metal traces. Fourier-transform infrared spectroscopy, x-ray photoelectron spectroscopy, focused-ion-beam and scanning-electron- microscopy were employed to determine any encapsulation material changes. *Main results.* No silicone delamination, passivation dissolution, or metal corrosion was observed during ageing. Impedances greater than 100 GΩ were maintained between the aluminium tracks for silicone encapsulation over SiO_*x*_N_*y*_ and SiC passivations. For these samples the only observed failure mode was open-circuit wire bonds. In contrast, progressive hydration of the SiO_*x*_ caused its resistance to decrease by an order of magnitude. *Significance.* These results demonstrate silicone encapsulation offers excellent protection to thin-film conducting tracks when combined with appropriate inorganic thin films. This conclusion corresponds to previous reliability studies of silicone encapsulation in aqueous environments, but with a larger sample size. Therefore, we believe silicone encapsulation to be a realistic means of providing long-term protection for the circuits of implanted electronic medical devices.

## 1. Introduction

Ensuring the longevity of implanted medical devices is critical for their clinical usefulness. This longevity is contingent on several factors, including extending the energy autonomy of such implants, usually by wirelessly transferring power, and, crucially, ensuring the robustness and reliability of the device. The current gold-standard for achieving this second goal is hermetically sealing the sensitive electronics in a water impermeable casing. The main limitation of this method is that it cannot be easily miniaturised. Very small package sizes are beyond the sensitivity of spectrometers to validate hermeticity and increasing the number of conductors and feedthroughs in the package is non-trivial [1, 2]. As an alternative, silicone encapsulation has previously demonstrated long-term protection of implanted thick-film electronic devices [3]. Despite this success, much of the current research into conformal device packaging is focused on parylene, liquid crystal polymers and novel inorganic coatings [4]. Such coatings are stiffer and more brittle than silicone, and often require an additional softer coating to better match the mechanical properties of soft tissue.

It is not obvious that a material as water-permeable as silicone would be useful but, for the years in which medical implants must remain functional in the body, all polymers are permeable to water vapour [5]. What is critical is that the encapsulant remains adhesively-bonded to the surfaces between the conductors so that there is no void into which the vapour can condense to form a conductive liquid film [6]. Some silicones are particularly suitable because they: (i) form chemical bonds to the surface that are hydrolysed slowly or not at all; (ii) have low modulus so that strains due to temperature change do not translate to high stress on the adhesive joints; (iii) have little ionic impurity; (iv) are impermeable to corrosion products; and (v) are biocompatible. These properties were investigated in a series of papers from PEK Donaldson in the 1990s [7–10] and N. Donaldson [11].

To our knowledge, the Finetech–Brindley sacral anterior root stimulator is the only device that has demonstrated reliability for a chronically implanted device protected only by non-hermetic conformal encapsulation. These devices have discrete components (diodes, resistors, capacitors and coils) encapsulated in a room temperature vulcanising (RTV) silicone encapsulant. Brindley [3] reviewed the performance of the first 500 of these devices, which were implanted in people with spinal cord injury, and found the mean time-to-failure was over 19 years, and that failures were usually not in the encapsulation but broken wires in the cables. These devices used thick-film technology with platinum-gold metallisation on alumina ceramic substrates. The endurance of the adhesion of the silicone to the alumina is excellent but the gap between metallised tracks is at least 2 mm.

The emerging generation of millimetre-sized implantable devices comes with stringent miniaturisation requirements, such that often dictate the use of thin-film interconnects, connecting electrodes to integrated circuits (ICs) and inherently within the ICs themselves [12–15]. The gaps between these conducting tracks can be more than 100 times closer than for the Finetech–Brindley implant, resulting in commensurately higher electric field strengths. Common thin-film passivations, such as silicon nitride, also have a relatively poor chemical resistance compared to the alumina substrates used for thick-film devices, dissolving when exposed to liquid water [16]. Consequently, the success of silicone encapsulation is not guaranteed for this new family of implantable devices.

The work described in this paper was done within the CANDO project (Controlling Abnormal Network Dynamics using Optogenetics), which aims to develop an optogenetic brain implant for treating intractable focal epilepsy. In the CANDO device, each electrode shaft inserted into the brain must have many conducting tracks. Our latest design has four LEDs and three electrodes on each shaft. Eleven tracks on a shaft width of 200 *µ*m requires track and gap widths of about 10 *µ*m, which is feasible for thin-film technology. These longitudinal tracks are the interconnects between the electrodes and LEDs on the shafts and the amplifiers and drivers in a custom-designed IC mounted at the end, at the surface of the brain. The thermosonically welded bonds to the LEDs and the wire bonds to the IC must be insulated, and our original design used adhesive silicone elastomers to encapsulate the shaft, the IC, the LEDs and their connections. Further technical details will be published in companion papers [17, 18]. For CANDO, we also require optical transparency (for transmission of LED light) and that two-part elastomers are used so that curing is possible in closed moulds. Crucially, the device must be continuously electrically-active in the brain and reliable for years, requiring validation of the silicone encapsulants.

Previous accelerated life tests using silicone encapsulants for implanted devices, or test structures representing implants, include the series described by Edell [19]. Thin-film platinum interdigitated combs (IDCs) and triple-track test structures with 20 *µ*m gap widths were patterned on SiO_2_ substrates and encapsulated in various silicones. Aged at 37 ^∘^C under a continuous 5 V bias, excellent adhesion between the silicones and SiO_2_ surfaces maintained insulation for up to 7 years in some samples. This provides a useful validation for the continued examination of silicone for modern devices with small feature sizes. However, the focus of these studies was to evaluate the insulation offered by silicone between exposed IC bond pads on encapsulated devices, not the corrosion of tracks in buried passivations which has also been observed during reliability studies of microelectronics [20]. Indeed, modern day devices may use materials more susceptible to degradation than the IDC materials evaluated by Edell. IC passivations, such as silicon nitride and oxynitride, will dissolve in condensed water [16]. Aluminium is commonly used for the tracks in thin-film technology, and yet it is prone to corrosion in the presence of Cl^−^ ions [21]. Therefore, it is still not obvious how long implanted devices would survive with continuous high electrical stress. Despite these concerns, a more recent ageing study of the Intracortical Visual Prosthesis from Illinois Institute of Technology showed no failures of four IC devices encapsulated in Dow 96-083 silicone aged at 121 ^∘^C for over 91 days [22]. As this is the only ageing study of a modern implant encapsulated in silicone that we are aware of, it is uncertain if these promising results can be expected for other encapsulated devices. Therefore, to confidently use silicone encapsulation for modern implants, we must conduct a more systematic investigation. This includes studying all expected failure mechanisms; the effect of various ageing conditions; and the use of different silicones, conductors and passivation materials.

In this study, we compared the ability of different silicones and passivation layers to prevent corrosion and insulate thin-film circuits intended for highly miniaturised implanted medical devices (table 1). IDC samples were aged up to 694 days in phosphate-buffered saline (PBS) at 67 ^∘^C with a continuous electrical bias. No metal corrosion was observed during this period, however maintenance of insulation was dependent on the characteristics of the passivation used. We show that the water permeability of polymer encapsulants is unimportant, provided adhesion is maintained and substrate materials block further moisture transport.

**Table 1.** A summary of the material comparison batches investigated. Batch A was abandoned because the silicone was not properly cured. WB = wire bond failures; IDC = failures in the interdigitated combs, as defined in the text.

					Failures	
Batch	Passivation	Silicone	*n* samples	Days	WB	IDC
A	SiO_*x*_	MED-6015	0	0	—	—
B	SiO_*x*_N_*y*_	MED-6015	12	694	3	0
C	SiO_*x*_	MED3-4013	9	610	1	8
D	SiO_*x*_N_*y*_	MED3-4013	4	562	1	0
E	SiO_*x*_N_*y*_ + SIC	MED-6015	13	581	4	0
F	SiO_*x*_N_*y*_ + SIC	MED3-4013	13	535	2	0

## 2. Methods

### 2.1. Sample preparation

#### 2.1.1. Fabrication of IDCs

The IDCs occupy a functional area of 24.8 × 4.0 mm, with each comb having 25 fingers (*N* = 50). They were fabricated in the London Centre for Nanotechnology. The width of the fingers and the inter-digit space is 20 and 60  *µ*m, respectively. A discussion on this geometry for sensing sample damage (e.g. delamination) is provided in the supplementary material (S1) (is available online at stacks.iop.org/JNE/18/055003/mmedia). Prior to fabrication, 525 *µ*m ± 25 *µ*m thick 10 cm diameter p-type single side polished silicon wafers (1–30 Ω·cm) with a thermally grown 1 *µ*m SiO_2_ on both sides were cleaned by 10 min of plasma oxygen treatment (4 mBar 300 W 13.656 MHz Diener Nano Plasma Unit). A bilayer positive photoresist process was used for photolithography. LOR$^{\textrm{TM}}$-10 B and Microposit$^{\textrm{TM}}$ S1818$^{\textrm{TM}}$ (MicroChem) were spin-coated at 4000 rpm then baked at 190 ^∘^C for 10 min and 115 ^∘^C for 1 min, respectively, achieving an overall photoresist thickness of approximately 6 *µ*m. Etch patterns were defined in the photoresist using a Quintel Wafer Mask Aligner, and subsequently developed in Microposit$^{\textrm{TM}}$ MF$^{\textrm{TM}}$-319 (MicroChem) for approximately 60 s. A 300 nm aluminium layer was deposited onto the surface using a Lesker PVD75 sputtering system. The sputtering chamber was pumped down to 10^−5^ mTorr as a minimum before sputtering at 5–50 mTorr in an argon atmosphere. To complete the lift off process, wafers were sonicated in Microposit$^{\textrm{TM}}$ remover 1165 (MicroChem) at 70 ^∘^C to strip the photoresist and unneeded aluminium. Wafers were then further cleaned by sonication in isopropanol (IPA), rinsed in deionised (DI) water and blown dry with N_2_ gas, and finally treated with oxygen plasma for 10 min to ensure complete removal of the photoresist.

The aluminium IDCs were coated with either SiO_*x*_, SiO_*x*_N_*y*_, or SiO_*x*_N_*y*_+SiC passivations. SiO_*x*_ is established in the semiconductor industry as both an interlayer dielectric and as a protective passivation layer [23]. SiO_*x*_N_*y*_ can maintain a similarly high resistivity as SiO_*x*_ and provides a better barrier to water and ions [24]. To further protect devices, we considered the deposition of a less soluble SiC layer on SiO_*x*_N_*y*_. The stability of SiC in aqueous environments is illustrated by the ageing studies of Cogan *et al* [25] and Hsu *et al* [26]. 1 *µ*m passivation layers of SiO_*x*_ or SiO_*x*_N_*y*_ were deposited via low-frequency plasma-enhanced chemical vapour deposition (PECVD) (138 Hz, 60 W) at a substrate temperature of 300 ^∘^C and pressure of 550 mTorr. SiO_*x*_ was deposited with a reactive gas mixture of SiH_4_ and N_2_O at flow rates of 12 and 1420 sccm, respectively. SiO_*x*_N_*y*_ was deposited with a reactive gas mixture of SiH_4_, N_2_O, and NH_3_ at flow rates of 40, 400, and 20 sccm, respectively. For SiO_*x*_ and SiO_*x*_N_*y*_ deposition, the total gas flow rate into the chamber was maintained at 1825 and 2420 sccm, respectively, with a mixture of He and N_2_ used as the carrier gas. Two fabricated wafers with a 1 *µ*m SiO_*x*_N_*y*_ passivation were given an additional 500 nm coating of PECVD SiC. SiC films were deposited in a PlasmaTherm Unaxis 790 Series PECVD system (13.56 MHz at RF power density of 0.20 W cm^−2^) at a substrate temperature of 325 ^∘^C and pressure of 1000 mTorr. SiC was deposited with a reactive gas mixture of SiH_4_ and CH_4_ at flow rates of 12 and 1420 sccm, respectively. The total gas flow rate into the chamber was maintained at 800 sccm using Ar as the carrier gas. Bond pads on the aluminium were exposed by a second photolithography step and reactive ion etching of the 1 *µ*m passivation layer. A dicing saw separated individual IDCs, which were cleaned by sonication in acetone (99.8%, Sigma Aldrich), IPA (99.5%, Fisher Scientific) and DI water (15 MΩ cm), for 5 min each.

#### 2.1.2. Manufacture of samples

**Figure 1.**
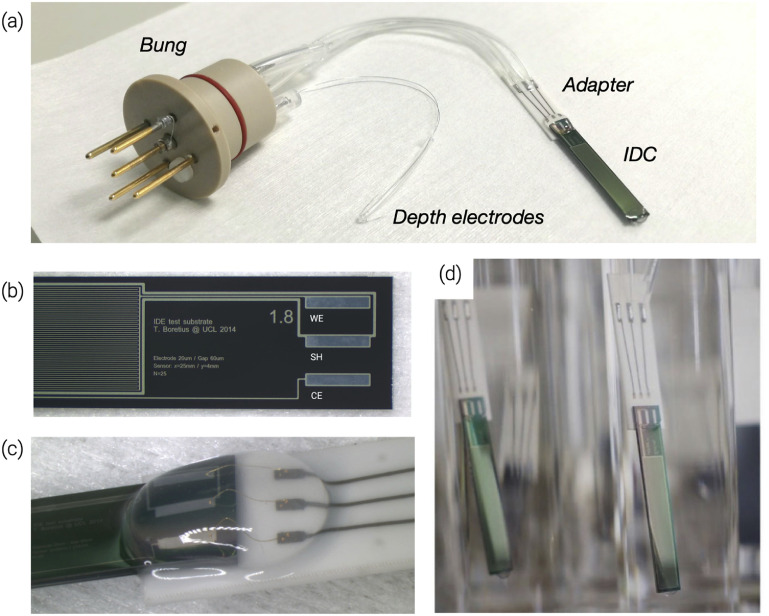
(a) An encapsulated IDC sample soldered to a polyether ether ketone (PEEK) bung with depth electrodes. (b) Diced IDC after fabrication with bond pad electrodes labelled as counter electrode (CE), shield (SH), and working electrode (WE) [27]. (c) IDC adhered and wire bonded to the adapter, with MED-1000 coated over the wire bonds and the entire structure encapsulated in MED-6015. (d) Manufactured IDCs immersed in PBS in borosilicate test tubes (Reproduced from [27]. © IOP Publishing Ltd. CC BY 3.0).

Manufactured samples are shown in figure 1. Screen printed ceramic adapters were used to facilitate electrical connections between the IDCs and the pins in the test tube bung (more details on the bung manufacturing process have been previously reported [27]). PtAu paste (ESL 5837) was screen printed onto 0.5 mm thick scribed alumina ceramic substrates (96% Coorstek grade ADS96R) and fired at 850 ^∘^C for approximately 1 h. A secondary glass sealing layer (ESL 4026A) was then printed over the PtAu tracks and fired at 500 ^∘^C for 5 h, leaving only the pads for wire bonding and soldering exposed. The proprietary sealing glass, likely a borosilicate glass, provides a better substrate for silicone adhesion than the PtAu metal tracks, reducing the risk of delamination occurring over the adapter. Prior to IDC attachment, the adapters were separated along their scribe lines and were soldered (Hydro-X multicore SnPb 60/40, water-soluble flux) to 75 *µ*m thick Teflon coated stainless steel wire. The adapters and solder joints were cleaned with a solution of 97 wt% DI water + 2.5 wt% Na_3_PO_4_ + 0.5 wt% multi-purpose detergent (Teepol$^{\textrm{TM}}$), followed by IPA and then DI water for 5 min in ultrasound each. Silicone tubes were slipped over the wires which, following encapsulation, allow for a continuous surrounding silicone layer from the IDC and adapter to the bung. Before the final assembly both adapters and IDCs were cleaned by sonication in acetone, IPA and DI water, for 5 min each. MED1000 RTV silicone adhesive (NuSil) was used to glue the diced IDC wafer pieces to the alumina adapters. Electrical connection between the two components was achieved with 30 *µ*m diameter thermosonically bonded gold wire bonds. The solder joints of the adapters and wire bonds were encapsulated with MED1000 (figure 1(c)). The assumption was that this silicone, marketed as an adhesive, would provide more reliable adhesion during ageing than the silicone encapsulants used for dip-coating the entire sample. Samples were then cleaned for 1 min sonication in acetone, IPA and then DI water, blown dried with N_2_ and further dried at 70 ^∘^C for at least 2 h. Prior to encapsulation, the samples were plasma activated with a Diener Zepto Plasma Unit with air plasma (21% ± 0.5% oxygen, balance nitrogen) for 10 min (4 mBar 100 W) then immediately encapsulated.

IDC samples were encapsulated in MED-6015 or MED-3-4013 (NuSil, Carpinteria, USA). We chose these two silicones after discussion with our local supplier of Nusil Medical Silicones (NuSil Carpinteria, USA). MED-6015, has a low viscosity (3800 cP) before cure and is optically clear, however it is not sold as an adhesive. Part A and B of MED-6015 were mixed using a SpeedMixer at 2500 rpm for 5 min. MED-3-4013 is a 2-part filled elastomer which is sold as an adhesive, however it is rather viscous (80 000 cP). Similar to the methods described in [28], we diluted the MED3-4013 pre-polymer in n-Heptane (99%, Fisher Scientific) to improve its viscosity and allow for dip-coating. An A:B:n-Heptane ratio of 1:1:2.4 was used, with the heptane first mixed with Part A at 2500 rpm for 10 min, then part B was added and spun at 2500 rpm until uniform consistency was achieved (which took approximately 10 min). Following complete mixing of the pre-polymers, encapsulation was achieved with a simple dip-coating procedure, where excess silicone was allowed to run off for 15 min, while the part was hanging (IDC downwards). Samples encapsulated in MED-3-4013 were left hanging overnight to allow all n-Heptane to evaporate from the pre-polymer. Curing for both polymers was completed at 150 ^∘^C for 15 min.

After curing, the stainless steel wires from the adapters were soldered to pins in test tube bungs (figure 1(a)). Two lengths of stainless steel wire were soldered to pins in the bung to make the PBS depth measurement electrodes (DE), which monitored evaporation of PBS in the test tubes [27]. The pins and base of the bungs were then encapsulated in Dow Corning 3140 RTV silicone.

Two types of control samples were manufactured alongside the IDC samples. *Bung* control samples consist of a bung with no sample, with the underside encapsulated in Dow Corning 3140. *Adapter* control samples were ceramic adapters manufactured as previously described and encapsulated in MED-6015 and wired to a test tube bung, but without an attached IDC. These control samples were used to identify potential leakage current pathways that are unrelated to the reliability of the encapsulated IDC.

### 2.2. Accelerated ageing

#### 2.2.1. Equipment

IDC samples were aged in our accelerated ageing and life-test apparatus (ALTA). In the ALTA each IDC sample is housed in an individual test tube filled with PBS (figure 1(d)), connected to a multiplexing printed circuit board (PCB, referred to as a *module*) to apply a voltage bias, and aged in a heated water bath. Details on the apparatus have been previously reported [27].

#### 2.2.2. Experiment protocol

Six different combinations (batches A–F) of MED-6015 or MED-3-4013 silicone encapsulants and SiO_*x*_, SiO_*x*_N_*y*_, or SiO_*x*_N_*y*_ + SiC passivation layers were investigated (table 1). Each ALTA batch contains 14 slots for samples. For this study 13 slots were used for IDC samples and one for a *Bung* control sample, which is used to monitor for leakage currents across the module PCB or bung. A batch of four *Adapter* control samples was used to assess the likelihood of leakage currents across the adapter.

All IDCs and control samples were aged at 67 ^∘^C with a 500 Hz biphasic 5 V voltage waveform (+5 V 500 *µ*s, −5 V 500 *µ*s, 0 V 1000 *µ*s), which imitates the CANDO LED drive waveform. Samples in the ALTA were biased continuously except during electrochemical impedance spectroscopy (EIS) measurements, which accounts for under 24% of the experiment duration (Donaldson 2018). Failure was defined as open circuit measurements or when impedances dropped below the expected range of measurement variance (determined by characterisation of *Adapter* control samples in section 3.1). Open circuit samples were removed from the apparatus. Ageing and EIS measurements were continued on failed samples with lowered impedances to observe if this effect preempted catastrophic failure later.

EIS was used to observe deterioration and failure of the samples (section 2.3.1). Measurements were performed prior to ageing and approximately each month after ageing commenced. Following failure, samples were removed from the ALTA. Post-mortem analysis with fourier-transform infrared spectroscopy (FTIR), focused ion beam (FIB), scanning electron microscopy (SEM), and x-ray photoelectron spectroscopy (XPS) (details in section 2.3) was performed on a selection of failed IDCs from batch C, D, and F that were decapsulated by soaking in DOWSIL DS-2025 overnight. Similar characterisation of unaged IDCs were used as controls. DOWSIL DS-2025 was ineffective at decapsulating MED-6015, with degradation of the silicone observed, but without lift off of the polymer from the substrate surface. As a result, it was not possible to perform a chemical analysis of the passivations encapsulated in MED-6015 (batch A, B, and E).

### 2.3. Characterisation

#### 2.3.1. EIS

EIS measurements were made with a Solartron Analytical Modulab XM, Potentiostat, Frequency Response Analyser and Femtoammeter. Measurements were performed between 10 mHz and 100 Hz using a 50 mV (RMS) applied sinusoidal voltage signal. Low frequencies better resolve high impedance resistive pathways indicating the beginning of failure, and 10 mHz was about the practical limit. Measurements were performed *in situ*, with the samples heated at 67 ^∘^C. The shield of the coaxial cable connecting the measurement module to the femtoammeter was held at ground potential, helping minimise interference for the low-current measurements.

#### 2.3.2. Imaging: SEM and FIB

Decapsulated IDCs were imaged with a Carl Zeiss XB 1540 SEM system. Samples were sent to Fraunhofer IZM for cross-sectioning, performed on a FEI Helios NanoLab 600i Dual Beam SEM with Focussed Ion Beam sectioning.

#### 2.3.3. FTIR

Attenuated total reflectance (ATR) FTIR characterisation was performed on a Shimadzu 8700 Fourier transform infrared spectrophotometer from 4000 to 400 cm^−1^ with a resolution 4 cm^−1^. FTIR absorption spectra were corrected in the Lab Solutions software using refractive indices for the SiO_*x*_ and SiO_*x*_N_*y*_ measured on a Horiba MM16 Spectroscopic Ellipsometer at 589 nm. The refractive index for SiO_*x*_N_*y*_ + SiC, received later in the project, was estimated as 3.0 [29] to correct the ATR measurements.

#### 2.3.4. XPS

XPS-measurement and depth profiles were carried out in a Quantera Hybrid from ULVAC-PHI (Q2). The measurements were performed using monochromatic AlK*α*-radiation (100 W) and a take-off angle Θ of 45^∘^. At this angle the information depth is approximately 7 nm. To eliminate crater edge effects, the data was acquired from a smaller region within the centre of the larger sputtered area. Therefore, a spot of 100 *µ*m was scanned over an area of 1200 × 500 *µ*m. First survey analyses were carried out during a test depth profile (sim86 nm size) to identify the elements present and the required depth for analyses. Then, accurate narrow-scans of Pt, C, Si, O, N and Al were measured for quantification in the depth profiles.

Concentration depth profiles were determined by alternating data acquisition cycles with sputter cycles, during which material was removed from the sample surface using an ion beam source. The sputter rate was calibrated using a reference SiO_2_ layer (sputter rate = 17.2 nm min^−1^).

#### 2.3.5. Statistical analysis of impedance changes and failure

The variance of measured impedance makes it difficult to identify when a change in impedance can be considered significant. To overcome this, significant changes to average batch impedances at 10 mHz and 10 Hz were identified by using the significance level (*p*) of 0.05, calculated by the *t*-test for an ordinary least squares regression (OLR) of the impedance measurements. The OLR was calculated using the Python Statsmodels library [30] on the logarithm (base 10) of the impedance measurements (*y* = 10^*mx* + *c*^). The 95% confidence interval and the two-sided 90% prediction interval were also calculated for the impedance regression.

## 3. Results

### 3.1. Control samples

**Figure 2.**
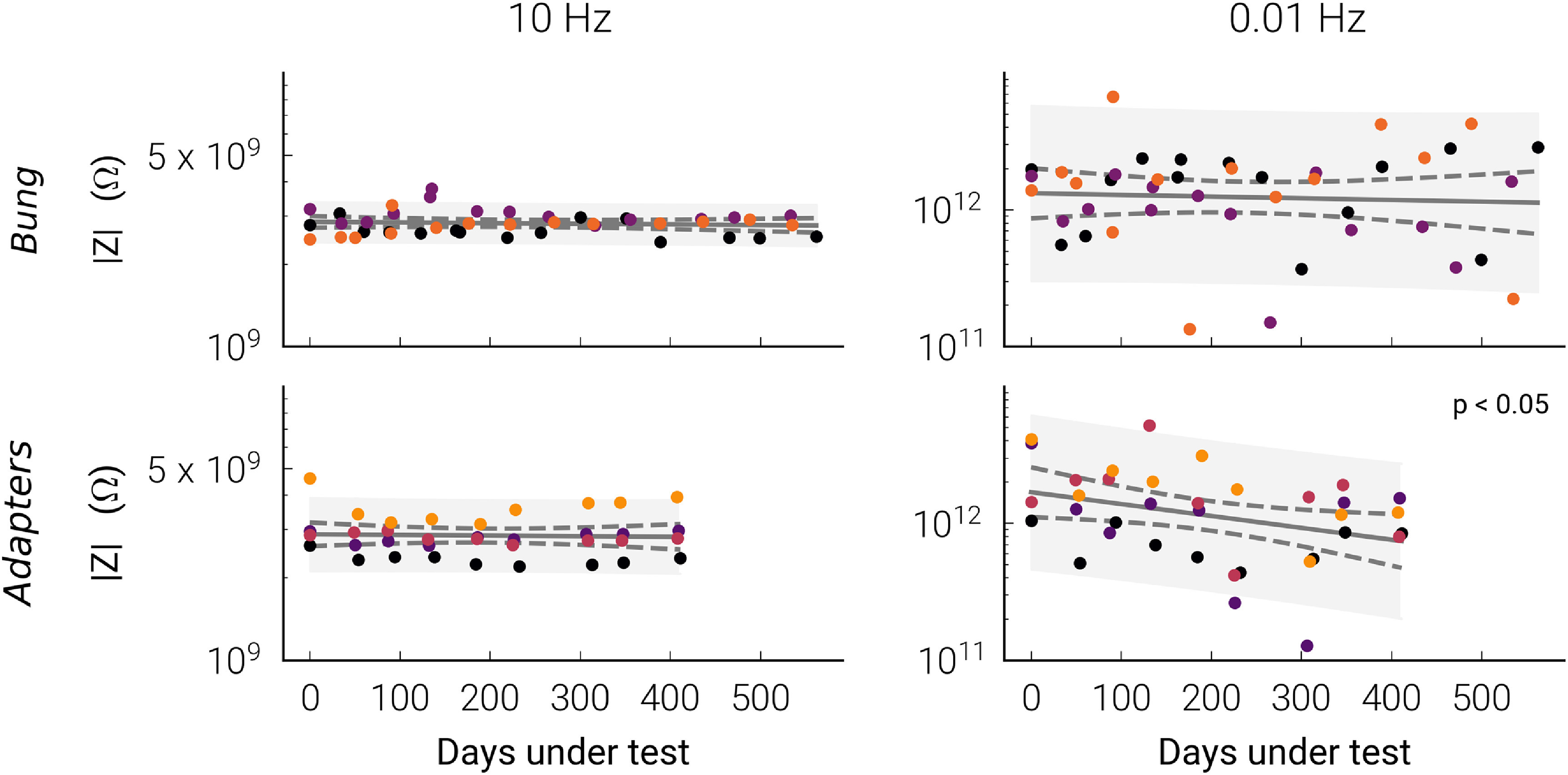
Impedance magnitude scatter plots and regressions for Bung (*n* = 3) and Adapter (*n* = 4) control samples. The different colours represent the four different control samples. The 95% confidence interval and 90% prediction interval is given by the dotted line and grey area, respectively.

An update to the design of the PCB module electronics caused measurement artefacts for two of the *Bung* control samples after the start of the experiment. These control samples could still be used to observe gross impedance changes across the PCB for their respective batches, but could not be included in the regression analysis. The *Bung* control sample for batch A was also excluded when this batch was removed from the experiment (see section 3.2.1). For the remaining three *Bung* control samples no significant decrease in impedance was found when aged for 560 days. For *Adapter* control samples, impedances at 10 Hz remained stable over 411 days of ageing, while a decrease (*p* = 0.038) in 10 mHz impedance from 1.69 to 0.74 TΩ was observed (figure 2). Prediction intervals show leakage resistances across the PCB module, bung, or encapsulated ceramic adapter of 10 GΩ or less at 10 mHz would be statistically unlikely. For the remainder of the experiment, ‘IDC failures’ were defined by impedances falling below this value, or open circuits.

### 3.2. Material comparison batches

#### 3.2.1. Manufacturing yield

Only preliminary results are available for batch A, as the first attempt of manufacture was unsuccessful due to defective curing of the MED-6015 silicone. Initial investigations did not identify an obvious cause, though ‘poisoning’ of two-part silicones can occur from a range of compounds that inactive the Pt catalyst needed for curing. The absence of this issue for the replacement batch, and all other batches in this experiment, suggests there was no fundamental issue in the cleaning process or material combinations used. The results from the defective batch and the preliminary results for the replacement samples are listed in the supplementary material (S2).

Each batch in the material comparison experiment ideally comprises 13 samples in two adjacent modules, however 100% yield was not achieved for three of the five remaining batches (table 1). The total number of samples for each experiment was determined by the number of functioning samples measured at room temperature (RT) after manufacture but before ageing. The majority of non-functional samples were due to faulty wire bonds (excessive force causing craters in the pad). These are referred to as ‘WB failures’ from hereon.

#### 3.2.2. EIS

**Figure 3.**
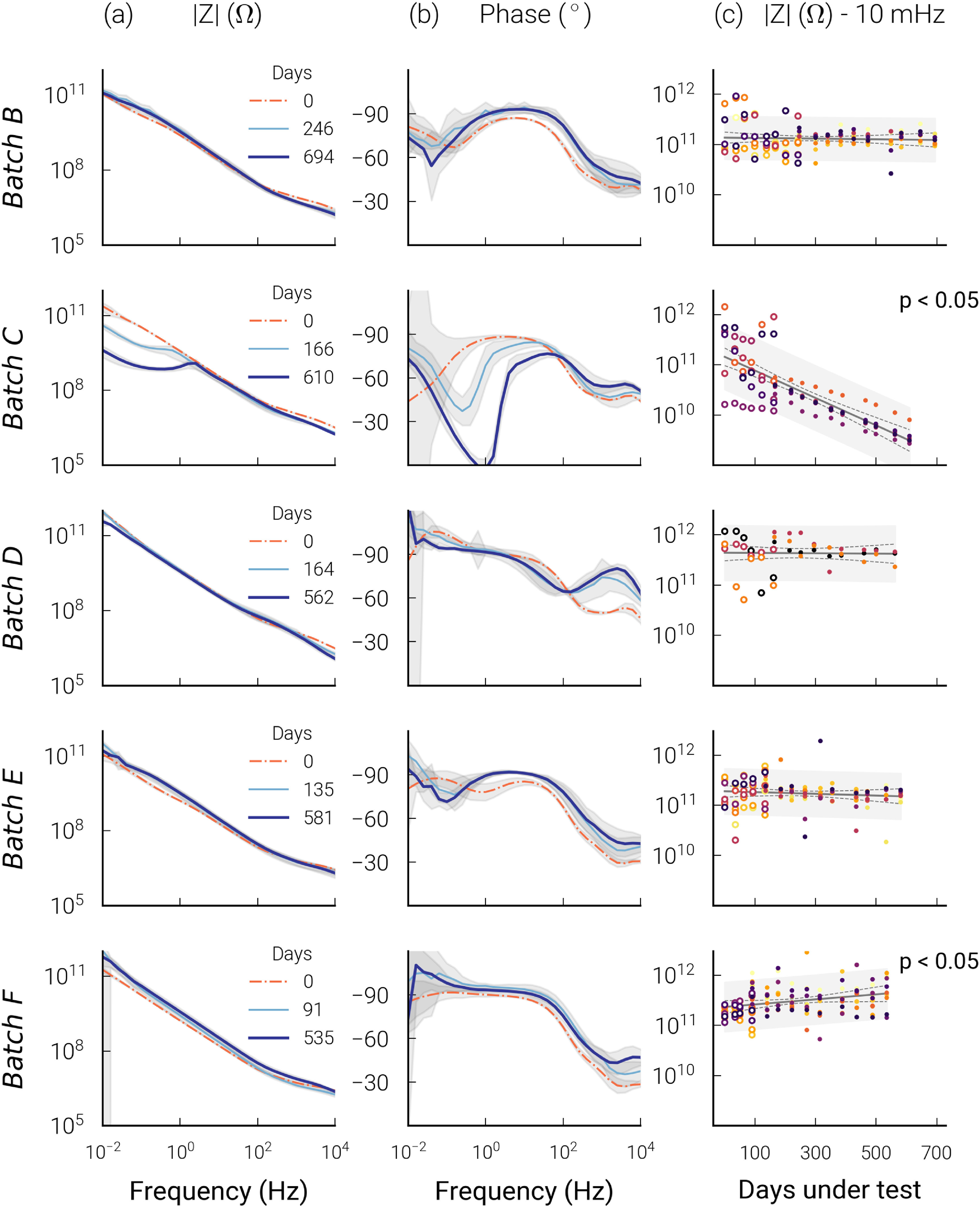
Average Bode (a) impedance magnitude and (b) phase plots for each batch’s impedance measurements over the duration of the experiment (± standard deviation shaded in grey). (c) 10 mHz impedance measurements and regressions for samples without WB failures. The different colours represent different IDC samples. Dashed lines and open faced markers represent corrected early measurements (detailed in S6). The 95% confidence interval and 90% prediction interval is given by the dotted line and grey area, respectively. Regressions with a significant change in impedance ($p \lt 0.05$) are noted.

Batches B, D, E, and F comprise IDCs coated with either SiO_*x*_N_*y*_ or SiO_*x*_N_*y*_ + SiC passivations in combination with MED-6015 or MED3-4013 (table 1). No significant impedance change was found for batches B, D, and E (figure 3), and measured impedances increased for batch F ($p \lt 0.001$) from 233 to 434 GΩ at 10 mHz. Average impedances for these four batches remained above 100 GΩ over the 535–694 days samples were tested. This result is consistent with the absence of observable delamination, void formation, or IDC corrosion in these batches (S3). In contrast, eight samples from batch C (SiO_*x*_ + 6015) failed, their impedances at 10 mHz falling below 10 GΩ. Average 10 mHz impedances decreased from 143 to 3 GΩ after 610 days ($p \lt 0.001$, figure 3). However, similar to the other batches, no observable degradation or corrosion was seen on microscopic examination. Preliminary tests of four unencapsulated IDCs, with only a single SiO_*x*_ (*n* = 2) or SiO_*x*_N_*y*_ (*n* = 2) passivation layer protecting the aluminium, failed within days, before repeated EIS measurements could be made. Corrosion was observed across the unencapsulated test structures (S4). Of the 51 samples tested from batches B–F, 11 suddenly failed from open circuit wire bonds (WB failures, S5). Changes in phase above 100 Hz was due to artefacts from the measurement apparatus, which have been extensively characterised in a previous publication [27].

#### 3.2.3. FTIR

**Figure 4.**
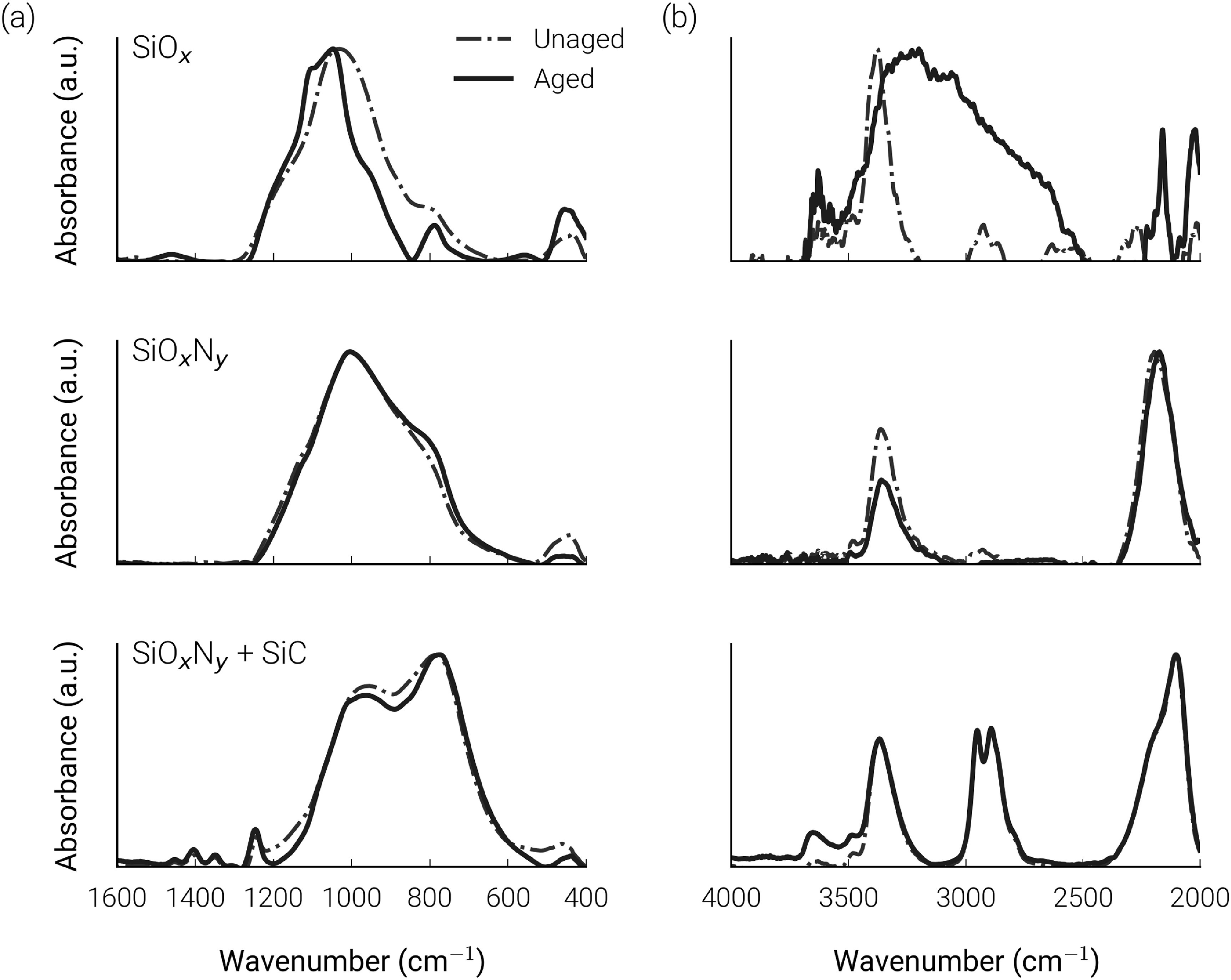
(a) High and (b) low wavenumber FTIR measurements of representative IDCs from batches C, D and F, stripped of silicone after ageing. Peaks are normalised with respect to their maximum to show the relative changes to the passivation materials.

The FTIR spectra of unaged and aged decapsulated IDCs for batch C (SiO_*x*_), D (SiO_*x*_N_*y*_), and F (SiO_*x*_N_*y*_ + SiC) are presented in figure 4. The unaged SiO_*x*_ spectra exhibited a relatively narrow peak around 1030 cm^−1^ and a shoulder at 1180 cm^−1^ from the symmetric and asymmetric stretching vibrations of Si–O, respectively. The peak at 3400 cm^−1^ is attributed to NH_2_, indicating nitrogen incorporation (discussed in section 4.2). The unaged SiO_*x*_N_*y*_ spectra similarly exhibited a Si–O peak at around 1000 cm^−1^ as well as a more significant shoulder at 850 cm^−1^ due to Si–N bonding. The higher nitrogen content of SiO_*x*_N_*y*_ resulted in a larger NH_2_ peak than observed for SiO_*x*_ and a large peak centred at 2200 cm^−1^ from Si-H. The measured spectra for the SiO_*x*_N_*y*_ + SiC bilayer comprised many of the same peaks observed for SiO_*x*_N_*y*_. A more significant shoulder at 800 cm^−1^ and peaks between 2800 and 3000 cm^−1^ were due to Si–C and C–H bonding in the material.

After 610 days of ageing at 67 ^∘^C, the batch C (SiO_*x*_) sample demonstrated a large change in FTIR spectra. The spectrum of the aged C sample more resembled that of SiO_2_, with a narrower Si–O peak shifted from 1030 to 1045 cm^−1^. Additionally, a broad peak between 3200 and 3700 cm^−1^ was observed after ageing. Only minor changes to the FTIR spectra were observed for batch D (SiO_*x*_N_*y*_, aged 562 days) and batch F (SiO_*x*_N_*y*_ + SiC, aged 535 days).

#### 3.2.4. XPS

**Figure 5.**
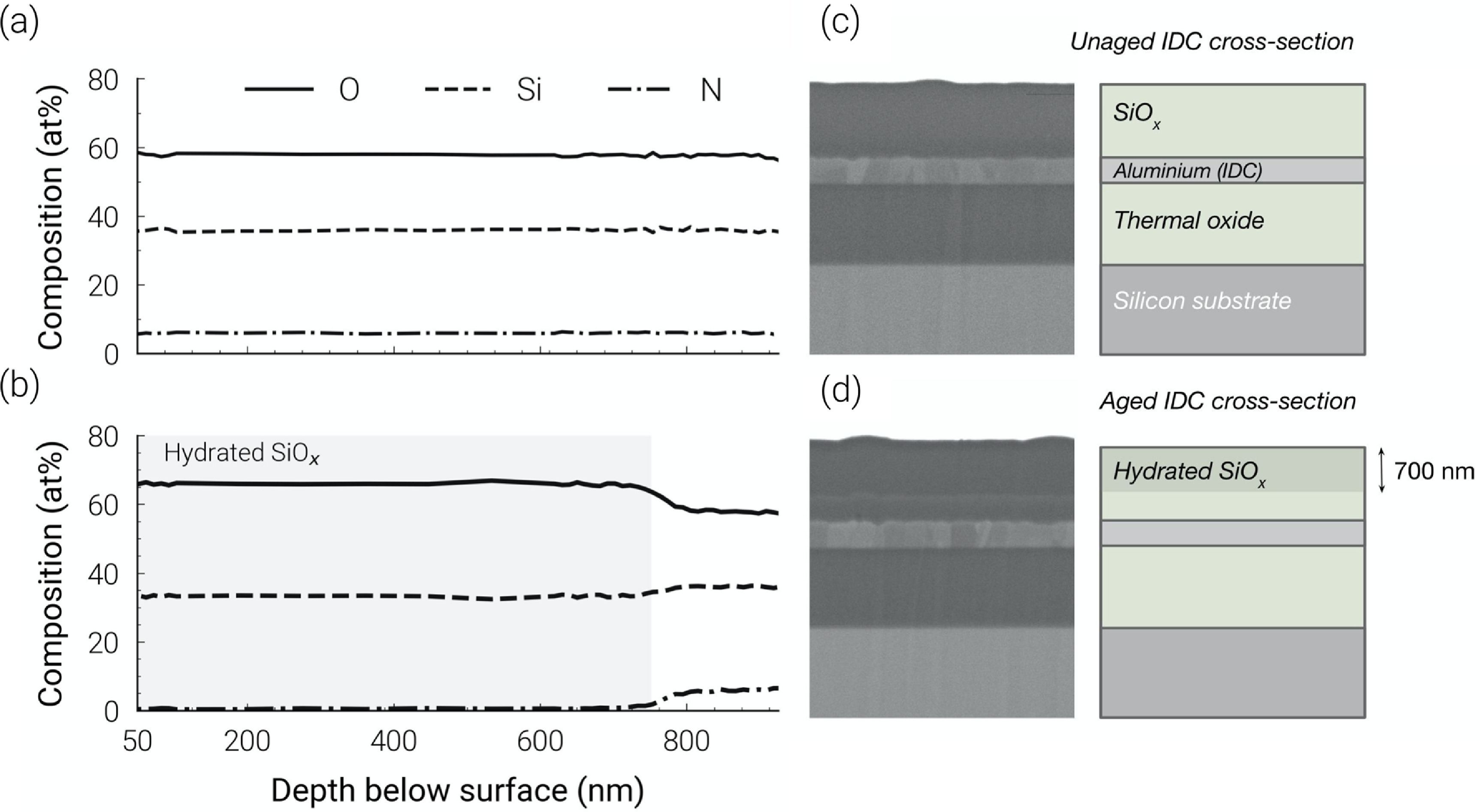
XPS depth profile of (a) unaged and (b) aged batch C sample (aged 570 days) after decapsulation. SEM+FIB cross sections of SiO_*x*_ samples at 30 000× magnification (left) and labelled illustration (right) for (c) unaged and (d) aged (570 days) IDCs (batch C, SiO_*x*_ passivation).

XPS analysis confirmed the as-deposited SiO_*x*_ to be non-stoichiometric, with a composition of approximately SiO_1.58_, which was constant across the depth of the passivation layer (figure 5(a)). Films had a nitrogen composition of 5 at%, which is consistent with the NH_2_ peaks observed in the FTIR spectrum. Depth profiling of a batch C sample aged for 570 days showed the top 700 nm of the SiO_*x*_ to be oxidised to SiO_2_, with the lower 300 nm remaining non-stoichiometric (figure 5(b)).

#### 3.2.5. FIB-SEM

FIB sectioning of the same unaged and aged SiO_*x*_ samples analysed in section 3.2.4 demonstrated degradation of the aged SiO_*x*_ passivation. After ageing, a darkened zone appeared in the upper 700 nm of the layer (figure 5(d)), matching the oxidised SiO_2_ depth measured by XPS section 3.2.4. No apparent change could be found for a batch D sample (SiO_*x*_N_*y*_), though this sectioned sample was aged for a shorter period before imaging (S7).

## 4. Discussion

The general aim of our experiments was to investigate whether silicone conformal coatings on passivated thin-film technology could be sufficiently reliable for chronic medical implants. To this end, we tested the insulation performance of the combined use of a passivation layer over the metal tracks, and silicone encapsulation. Tailored test structures were aged at 67 ^∘^C with a ±5 V stress, giving an electric field strength of 8 kV m^−1^. Samples were aged for 535–694 days and in four of the batches (B, D, E & F) no corrosion or insulation failures occurred (table 1). These very promising results were for SiO_*x*_N_*y*_ passivation, with or without overlying SiC, and either medical grade silicone used in this study (MED-3-4013 or MED-6015).

The biphasic ageing voltage represents the drive waveform to the LEDs in our optogenetic implant application. It is biphasic, with a reverse phase following the forward phase which causes light emission, to reduce the risk of electrolytic effects from leakage currents. A more severe test of the insulation is a continuous or ‘DC’ bias [31]. We extended the experiments described here to DC and with batches at other temperatures to estimate the endurance at body temperature, and these will be described in future papers.

### 4.1. Encapsulation of SiO_*x*_N_*y*_ and SiC passivations

Ten failures were recorded from the 42 samples in batches B, D, E, and F. All of these were due to open circuit wire bonds. No leakage currents or other impedance changes were observed for samples before wire bonds went open circuit, suggesting the silicone maintained insulation between the bonds. The majority of wire bond failures were clustered towards the beginning of the experiment and were attributed to poor wire bonder settings resulting in weak Al–Au welds (S5). The lack of significant impedance decrease for the surviving samples, combined with the absence of corrosion and IDC degradation, indicates that the silicones tested maintained adhesion to both the SiO_*x*_N_*y*_ and SiC passivation layers. Furthermore, only relatively minor differences in the FTIR spectra were observed for the SiO_*x*_N_*y*_ (batch D) and SiO_*x*_N_*y*_ + SiC (batch F) passivations after ageing (562 and 535 days, respectively). Due to the excellent reliability of the SiO_*x*_N_*y*_, no improvement could be discerned from the additional SiC coating given to the IDCs in batches E and F. Because of the improved chemical resistance of SiC compared to the conventional dielectrics we presume that, had the SiC samples been aged for longer, reliability differences between these batches may have become observable [25]. Early in the project preliminary testing of unencapsulated IDCs protected only by SiO_*x*_ or SiO_*x*_N_*y*_ showed rapid failure and corrosion. These findings correspond to previous studies, which have shown PECVD passivation materials (SiO_*x*_, SiO_*x*_N_*y*_, SiN_*x*_) dissolve in water [16, 32, 33], and that this may be accelerated under bias [20]. Therefore, we are confident the excellent reliability achieved in these batches can be attributed to the additional silicone encapsulation. A comparison of these results to similar encapsulant studies is given in section 4.3. Curiously, impedances increased for batch F, which was attributed to a measurement artefact of the ageing apparatus. The cause of this artefact is outside the scope of this article and has been investigated previously [see 34, chap 7]. Measurements of batch F samples temporarily removed from the ALTA confirmed this artefact did not mask any impedance changes of interest in this study.

### 4.2. Deterioration of SiO_*x*_ samples

FTIR and XPS characterisation of an unaged sample also showed our ‘SiO_*x*_’ films to contain 5 at% nitrogen. This was attributed to the use of N_2_O as a gas reactant and N_2_ as a carrier gas during deposition, and was similarly observed by Ay and Aydinli [35]. Despite the positive results observed for batches B, D, E, and F, samples from batch C demonstrated clear changes to their chemical and electrical characteristics. After 610 days of ageing, average 10 mHz impedance measurements decreased by over an order of magnitude. Though no corrosion was observed for this batch either, this impedance difference is striking. To use silicone encapsulants for chronic implants with confidence, we must understand the cause of this change.

#### 4.2.1. Potential degradation mechanisms for SiO_*x*_ samples

**Figure 6.**
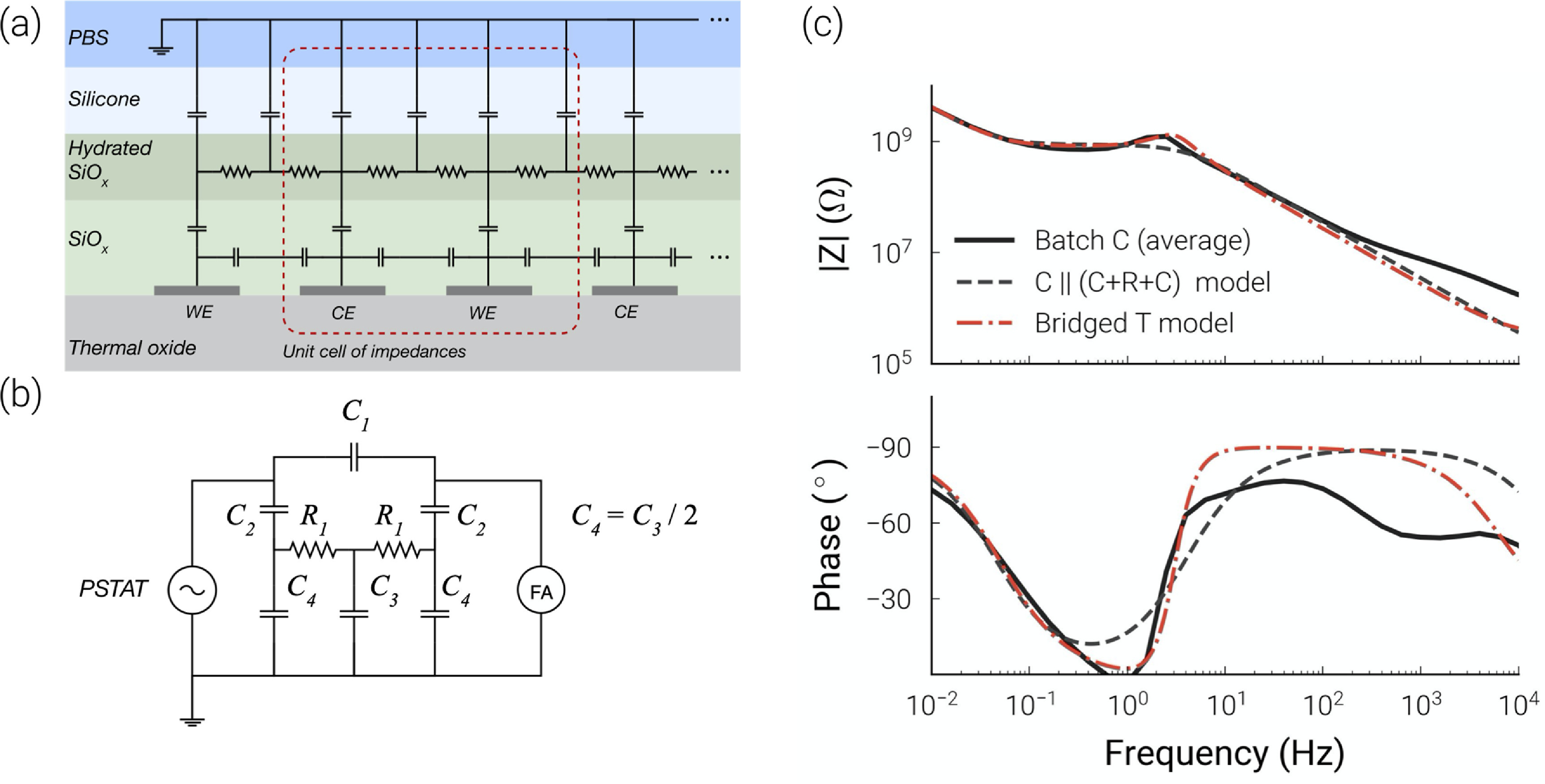
(a) Cross section with impedances pathways of IDC with hydrated passivation. (b) Equivalent circuit with PBS grounded (Bridged-T model). (c) Fits of the C $||$ (R+C) and Bridged-T model to average batch C measurements (values given in table 2). Models were fitted to the measurements between 10 mHz and 100 Hz.

An expected failure mode of the IDCs was delamination of the encapsulation, leading to water condensation, followed by dissolution of the passivation and corrosion of the metal [36]. As the IDC designs employed in this study can sense delamination of the silicone encapsulation (S1) it is reasonable to first consider this as an explanation of the observed impedance changes. Delamination of silicone can often be observed by a silvery film of water condensed below the encapsulant, which was not found for any of the IDCs in batch C, suggesting no delamination occurred. Additionally, drying aged IDCs for 4 days at 90 ^∘^C did not greatly reduce the leakage resistance (S8), suggesting that the impedance decrease during ageing was not primarily due to liquid water condensed between the SiO_*x*_ dielectric and delaminated areas of silicone. As discussed in S1, delamination may be electrically modelled as a C $||$ (C+R+C) equivalent circuit, giving a maximum capacitance increase of 214 pF. This is far lower than the changes observed, and the equivalent circuit model fits the data poorly (figure 6(c)), which is considered further in section 4.2.2.

**Table 2.** Components fit to the average batch C measurement using the C $||$ (C+R+C) and Bridged-T models.

C $||$ (C+R+C)		Bridged-T	
Component	Value	Component	Value
*C*_1_	45.8 pF	*C*_1_	59.1 pF
*C*_2_	8002.8 pF	*C*_2_	8040.3 pF
		*C*_3_	293.2 pF
		*C*_4_	146.6 pF
*R*_1_	882.3 MΩ	*R*_1_	388.9 MΩ

An alternative theory for the observed impedance decrease is that the SiO_*x*_ characteristics have changed because of water vapour permeating through the outer silicone layer. Indeed, the diffusion of water through silicone encapsulants has been shown to cause failure of anisotropic conductive adhesives for back-end packages during reactive ageing studies [37]. The shift of the FTIR Si-O peak and the formation of a Si–OH peak at 3200–3700 cm^−1^ are indicative of moisture induced oxidation of Si–Si bonds in the non-stoichiometric SiO_*x*_ [38–40]. After ageing, the SiO_*x*_ became stratified into two regions along the height of the layer. XPS depth profiling demonstrated the upper region to be completely converted to stoichiometric SiO_2_, with the nitrogen composition falling to 0%. Lee *et al* [41] observed a similar top-down conversion of low-temperature PECVD SiO_*x*_N_*y*_ to SiO_2_ in pressure cooker tests, proposing that interstitial diffusion, nano voids and defects allowed the permeation of moisture into the films. Desorption of nitrogen then proceeded by the expected reaction between water and Si–N, releasing ammonia and forming SiO_2_ [16, 40, 41].

The mechanism for conduction in the batch C samples samples may be similar to that of hydrated silica gel. Dissociation of Si–OH and adsorbed water form mobile protons (H_3_O^+^) that facilitate surface conduction through the porous network of the material [42, 43]. Temperatures between 190 ^∘^C–355 ^∘^C are required to remove surface bound water [44, 45]. Therefore, the reduced impedances observed in SiO_*x*_ samples, even after extensive drying at 90 ^∘^C, are consistent with conduction from adsorbed water species in the film. The absence of degradation and conduction for the SiO_*x*_N_*y*_ and SiC materials used here supports the notion that these typically denser films can better prevent moisture permeation and maintain insulation when aged in aqueous environments.

#### 4.2.2. Modelling degradation of SiO_*x*_

**Figure 7.**
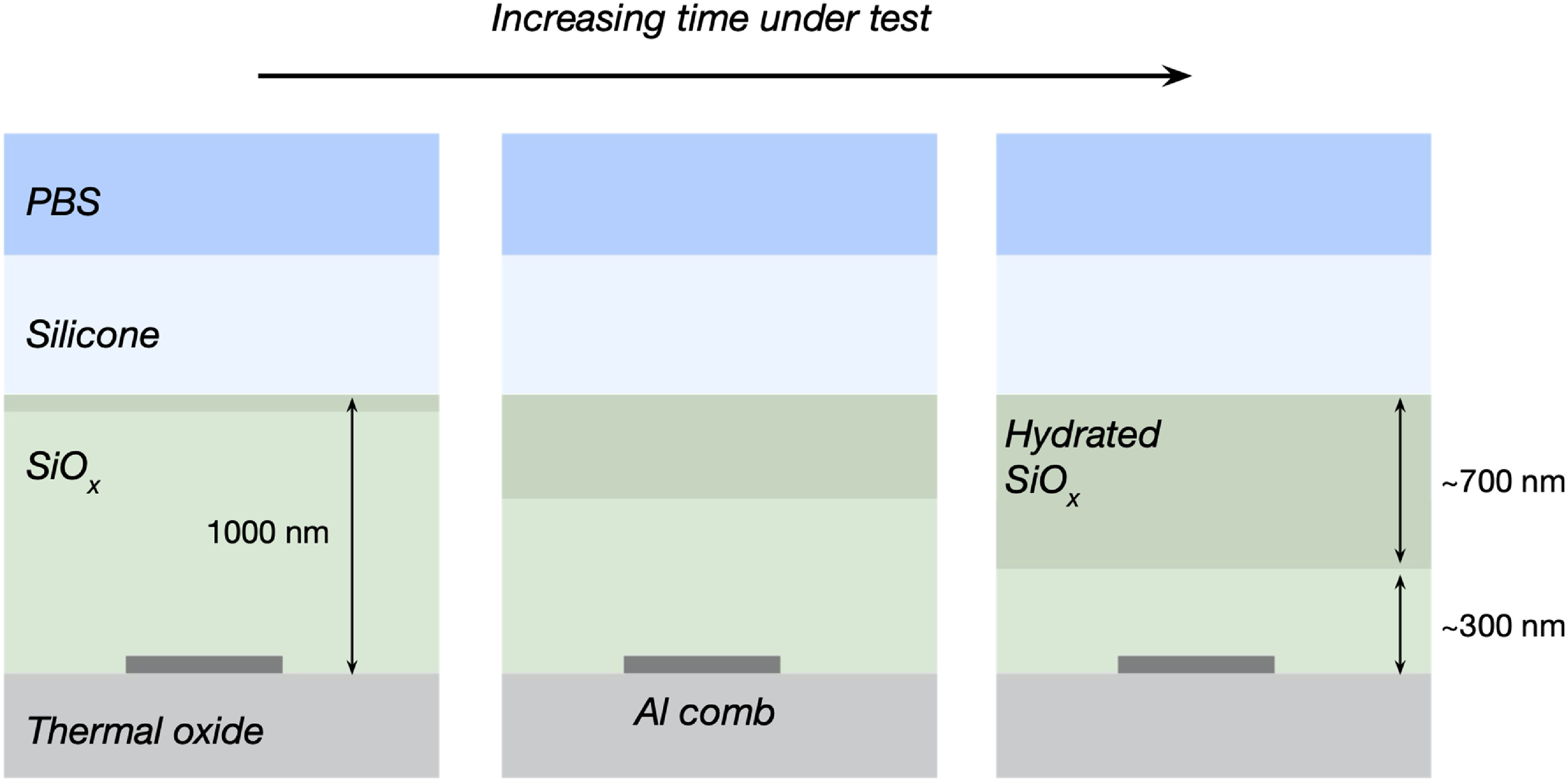
Diagram showing the progressive hydration of the SiOx layer. The capacitance between the metal track and the conductive layer increases as the unhydrated layer gets thinner.

An illustration of the impedance pathways for an IDC with a weakly-conductive hydrated SiO_*x*_ is given in figure 6(a). It is possible to simplify this to a more manageable equivalent circuit. If we ignore the fringing effect of electrode fingers at the very edge of the combs, the impedance across the IDC is approximated by *N*/2 unit cells in parallel, where *N* is the total number of tracks in the IDCs (*N* = 50 here). By combining the parallel and series components, the description of an IDC can be distilled to what is referred to as the Bridged-T model (as it incorporates a Bridged-T filter). A more detailed derivation of this model is given in S9. This model replicates the EIS characteristics of aged batch C samples, having the magnitude peak, observed around 2 Hz, and rapid change in phase seen below this frequency (figure 6(c)). By fitting the Bridged-T model to the average EIS results of batch C (excluding samples with WB failures), we can ascertain estimations for the component values (table 2, the fitting procedure is described in S6). C_2_ represents the vertical capacitance across the remaining undamaged passivation, which will increase as this region thins from further moisture absorption (figure 7). In this way, the proposed model can explain the large capacitance changes observed for batch C.

While no corrosion was observed for samples using a SiO_*x*_ passivation, clearly this material combination with silicone encapsulation is unsuited to providing long term insulation and protection to buried metallisation; eventually all the passivation will become hydrated and it seems likely that the metal would then corrode. The results presented here indicate that the other passivation layers are superior because they do not adsorb water by hydration. However, in the duration of this experiment, we could not see any superiority in SiC over SiO_*x*_N_*y*_.

### 4.3. Comparisons with other encapsulants

A useful comparison to our results is given by a recent review by Ahn *et al* [4] of over 21 encapsulation reliability studies. The most promising results presented were from multi-layer coatings that incorporated atomic layer deposited (ALD) aluminium oxide (Al_2_O_3_) or hafnium oxide (HfO_2_), neither of which demonstrated a change in insulating properties when aged at temperatures above 80 ^∘^C in PBS for 6 months [46, 47]. Due to the lower ageing temperatures imposed in our study, it is difficult to estimate equivalences without relying heavily on crude temperature-acceleration relationships. However, for the four batches (B, D, E, and F), the extended period of testing (535–694 days) and the absence of failures (excluding open circuit wire bonds) would indicate silicone-passivation encapsulation to be just as effective in preventing thin-film corrosion as these other encapsulation technologies. This is further evidenced by previous work from our group, which demonstrated only a single encapsulation failure for 36 foundry-processed IDC structures coated in MED-6015 silicone, aged under a constant 5 V DC bias for over 302 days at between 47 ^∘^C and 87 ^∘^C [48]. In fact, based on the knowledge we gained from the study presented here and the results in Jeong *et al* [47], we have also recently investigated the use of silicone as encapsulation for ALD HfO_2_ passivated thin-films. RT soak investigations showed stable impedance results over the 450 days of the study, indicating, at least in these conditions, an excellent adhesion between the silicone and HfO_2_ and good stability of the ALD [49].

Though Parylene-C is often investigated in the context of non-hermetic conformal packaging of implants, it has a relatively high modulus and is brittle, with encapsulation failures observed from cracks or blisters formed during *in vivo* and *in vitro* ageing [50–52]. A recent multiyear *in vivo* study also demonstrated previously unreported degradation of parylene-C implants [53]. In comparison, silicone encapsulation is soft and tough, and can withstand decades of implantation without significant changes to its mechanical properties [54].

### 4.4. A review of pre-encapsulation cleaning methods

The findings of this study demonstrate that excellent corrosion protection is achieved with a passivation/silicone bilayer encapsulation. However, this requires the silicone to remain adhered to the passivation layer, which is strongly dependent on its cleanliness. To give some explanation to the positive results achieved, and a useful resource for those hoping to replicate this process, we now provide more context to our cleaning procedure.

The presence of contamination, due to inadequate cleaning or processing, will prevent the encapsulant from bonding to the surface, thereby creating a void in which water may condense, initiating the timeline of failure. Extensive characterisation of microelectronics encapsulation confirms surface cleanliness to be a primary factor affecting encapsulant adhesion and device reliability [36]. Such contamination on the surface of a pre-encapsulated device will likely be both ionic and organic, and can arise from various sources (flux, fingerprints, spittle, etc). The different properties of these contaminants necessitates multiple cleaning processes to ensure a contaminant-free surface. An effective approach to cleaning is to combine a solvent clean, utilising polar and non-polar solvents, and a plasma or oxidation clean, which can breakdown residual organics. For the current study, prior to encapsulation, samples were cleaned by sonication in acetone, IPA, and then DI water, and, after drying, were further cleaned in an air plasma.

Edell [19] similarly employed a solvent clean of acetone, IPA, and DI water when evaluating the adhesion strength of silicone to SiO_2_ surfaces aged in saline. Using these three solvents, in combination with a pirhana etch and UV-Ozone clean, they were able to achieve excellent adhesion between platinum-catalysed addition-cure silicones and SiO_2_ surfaces, as tested by long-term ageing studies of encapsulated IDCs over several years. However, they reported that this solvent clean alone was not always sufficient to remove accumulated contamination on samples after storage. Ianuzzi [55] identified the use of O_2_ plasma (300 W) to be the most crucial step in their cleaning process, measured by the reduction in device failure for any of the cleaning steps tested individually. Plasma cleaning creates a gas of ionised particles that bombard and react with organic contaminants on the device surface. These are vaporised to CO_2_ and H_2_O, which can be removed by the chamber’s vacuum pump. This process is safe for aluminium metallisation, with little to no observable increase in the Al oxide layer following cleaning [55, 56]. Other wet oxidation processes used to remove organics, such as a pirhana etch, employ strong acids and are not suitable for aluminium. For the current study, IDCs were cleaned with air plasma at 100 W for 10 min. The use of air plasma, as opposed to pure O_2_ makes it difficult to validate the current process from the results of Iannuzzi [55], however we think the ionic bombardment is a key factor in the plasma cleaning process, which can be achieved with Ar, O_2_, N_2_ or air plasma [57]. Even mild processes (Ar at 40 W for 1 min) have been shown to dramatically reduce carbon contamination on metal surfaces [58]. We suggest that researchers designing processes for implant manufacture and encapsulation should follow the same protocol applied here, perhaps with modification to the plasma clean if justified by the literature or empirical evidence.

### 4.5. Current and future ageing tests

Often following accelerated ageing tests an estimation of lifetimes is given. A simple approach is to state the mean time to failure (MTTF) at 37 ^∘^C, calculated by assuming the rate of failure doubles with every 10 ^∘^C increase in testing temperature. Such an estimate is not provided here as we believe this approximation too crude for a quantitative statement of reliability. Additionally, the MTTF is not a useful indicator of the relevant lifetime of a medical implant, as it could very well correspond to the point at which more than half of devices would have failed (depending on the distribution of failures). Some authors report a more detailed analysis of the results of accelerated ageing studies [59] and we plan to detail a more considered statistical approach to calculating lifetimes in a future publication. We also plan to investigate ageing solutions that incorporate reactive oxygen species, as these have been shown to generate more realistic ageing effects for other encapsulants [53]. For now, we may state that, given the positive findings for four of the five batches in this study, combined with previous demonstrations of corrosion protection of silicone encapsulated microelectronics and medical implants, future tests should be expanded to: (i) *in vivo* ageing studies, to ensure the results observed are representative of the stresses on implanted devices; and (ii) failure modes other than interconnect corrosion. Reliability studies of encapsulated microelectronics typically accelerate failures of devices under humid conditions, such as a temperature humidity bias test, and can provide evidence of possible failure modes for devices running under implanted conditions (i.e. 100% relative humidity). From a review of this literature, the failure modes dependent on moisture and ionic contamination are bond pad corrosion [20, 60–62] and MOSFET threshold voltage shifts, which may be caused by damage to the gate oxide from ionic contamination or water-enhanced charged-carrier injection [63–66].

Importantly, there is limited information that can be gained from this study regarding these other failure modes, necessitating additional ageing studies that are specifically designed to characterise these phenomena. Different failure modes may not share the same temperature acceleration relationship. Importantly, both ionic contamination and charge injection of MOSFET gate oxides exhibit an inverse temperature acceleration factor [67]. Though this relationship only considers contamination already present at the gate oxide and does not consider the role of temperature in accelerating diffusion of such species from the external environment. The authors have recently introduced a new platform fully integrated on a CMOS IC, which can be used to detect potential ingress of moisture/ions through the ICs metal layers [68]. This ingress will manifest itself in the form of changes in the resistance of the interlayer dielectrics, which can be picked up by the platform. Such a tool, when used in new variable temperature studies, could provide further insight regarding the role of temperature in diffusion processes.

The above points highlight that accelerated ageing tests at elevated temperatures, as performed here, will not uniformly accelerate all failure modes, leading to inaccurate estimations of total device reliability. It is for this reason that the discussion and conclusions of this study are framed from the perspective of only considering metallisation corrosion and insulation of implanted devices. Future studies will be designed to address other relevant failure mechanisms. To maximise the utility of such ageing studies, we encourage others researching device encapsulation to adopt a similar considered approach to their reliability investigations.

## 5. Conclusions

This study presented a thorough examination of a promising non-hermetic conformal approach based on silicone to protect passivated thin-film circuits intended for miniaturised implanted medical devices. No metallisation corrosion was observed for any of the encapsulated IDCs tested in this study, which employed SiO_*x*_, SiO_*x*_N_*y*_ and SiO_*x*_N_*y*_ + SiC passivations and MED-6015 and MED3-4013 encapsulants. Therefore, for the 535–694 days of testing, all material combinations satisfactorily protect against corrosion for our *in vitro* experiment. EIS measurements help detect degradation of the coating materials that may indicate the future risk of corrosion, such as silicone delamination. No significant change in impedance was noted for the SiO_*x*_N_*y*_ and SiO_*x*_N_*y*_ + SiC passivations when combined with either MED-6015 and MED3-4013. Therefore, under the conditions tested, no difference in the protection offered by SiO_*x*_N_*y*_ and SiO_*x*_N_*y*_ + SiC passivations or MED-6015 and MED3-4013 silicones can be stated. However, EIS measurements illustrated the poor performance of using PECVD SiO_*x*_ in combination with silicone rubber as a means of insulating implanted electronics. We present evidence that moisture absorption by the SiO_*x*_ layer created a weakly-conductive region, without delamination of the silicone. This is supported by electrical, chemical and physical characterisation of the layers, with an electrical model giving a reasonable fit for the characteristic impedance spectrum. This mode of degradation appears intrinsic to the SiO_*x*_, independent of surface contamination and delamination. This result indicates that maintenance of insulation is dependent on the characteristics of the passivation used. This highlights that failure of non-hermetic packages will not necessarily be from gross electrochemical degradation of the metallisation, and that all possible failure modes need to be investigated before confirming the suitability of an encapsulant, especially if it is to protect implanted active CMOS chips.

A takeaway from this study is that, while silicone is permeable to water vapour, this is unimportant as long as there is no loss of adhesion to its substrate and that this substrate is itself a good water barrier. A thorough cleaning procedure, followed by a simple dip-coat of silicone over commonly used passivations (SiO_*x*_N_*y*_ or SiO_*x*_N_*y*_ + SiC) created a bilayer that prevented corrosion of thin-film circuits at least as effectively than that previously reported in the literature. Validation of these materials to prevent other possible failure modes, combined with complementary *in vivo* studies, will provide strong evidence that conformal encapsulation is a realistic approach for protecting chronically implanted active devices.

## Data Availability

All data that support the findings of this study are included within the article (and any supplementary files).
